# Covid-19 pandemic lessons: uncritical communication of test results can induce more harm than benefit and raises questions on standardized quality criteria for communication and liability

**DOI:** 10.1080/21642850.2021.1979407

**Published:** 2021-09-21

**Authors:** Franz Porzsolt, Gerit Pfuhl, Robert M. Kaplan, Martin Eisemann

**Affiliations:** a Institute of Clinical Economics (ICE) e.V., Ulm, Germany; b UiT The Arctic University of Norway, Tromsø, Norway; c Stanford University, Stanford, CA, USA

**Keywords:** COVID-19 pandemic, risk perception, risk communication, information intervention, decision-making

## Abstract

**Background:**

The COVID-19 pandemic is characterized by both health and economic risks. A ‘safety loop’ model postulates risk-related decisions are not based on objective and measurable risks but on the subjective perception of those risks. We here illustrate a quantification of the difference between objective and subjective risks.

**Method:**

The objective risks (or chances) can be obtained from traditional 2 × 2 tables by calculating the positive (+LR) and negative (−LR) likelihood ratios. The subjective perception of objective risks is calculated from the same 2 × 2 tables by exchanging the X- and Y-axes. The traditional 2 × 2 table starts with the hypothesis, uses a test and a gold standard to confirm or exclude the investigated condition. The 2 × 2 table with inverted axes starts with the communication of a test result and presumes that the communication of bad news (whether right or false) will induce ‘Perceived Anxiety’ while good news will induce ‘Perceived Safety’. Two different functions (confirmation and exclusion) of both perceptions (Perceived Anxiety and Safety) can be quantified with those calculations.

**Results:**

The analysis of six published tests and of one incompletely reported test on COVID-19 polymerase chain reactions (completed by four assumptions on high and low sensitivities and specificities) demonstrated that none of these tests induces ‘Perceived Safety’. Eight of the ten tests confirmed the induction of ‘Perceived Anxiety’ with + LRs (range 3.1–5900). In two of these eight tests, a −LR (0.25 and 0.004) excluded the induction of ‘Perceived Safety’.

**Conclusions:**

Communication of test results caused perceived anxiety but not perceived safety in 80% of the investigated tests. Medical tests – whether true or false – generate strong psychological messages. In the case of COVID-19 tests may induce more perceived anxiety than safety. Risk communication has to balance objective and subjective risks.

## Introduction

In February 2020 it became clear to epidemiologists that the new SARS-CoV-2 had traveled around the globe, and subsequently on 11 March 2020 the WHO classified COVID-19 as a pandemic (WHO, 2020). 16 months later, the COVID-19 pandemic continues to advance in many countries and is far from over. In the absence of reliable data and tools, politicians had to make decisions based on limited information. Accordingly, there is considerable variation by country on strategies to control the number of infected persons. Some countries imposed complete lockdowns whereas other countries issued public health advice and partial lockdowns. Compliance with public health advice rests on a citizen's subjective probability of contagion, the subjective assessment on the noxiousness of contagion, and the confidence in the real-world effectiveness of the recommended interventions (Rogers, 1975). This Protection Motivation Model thereby emphasizes the importance of perceived over objective risks. For example, the higher the perceived efficacy of imposed COVID-19 restrictions and perceived efficacy of one's own protective behavior the better was the mental health among participants (Mækelæ et al., 2020). High perceived risk of contagion, on the other hand, can reduce prosocial behavior, dubbed the fatalism effect (Abel, Byker, & Carpenter, 2021; Akesson, Ashworth-Hayes, Hahn, Metcalfe, & Fatalism, 2020).

**Figure 1.**
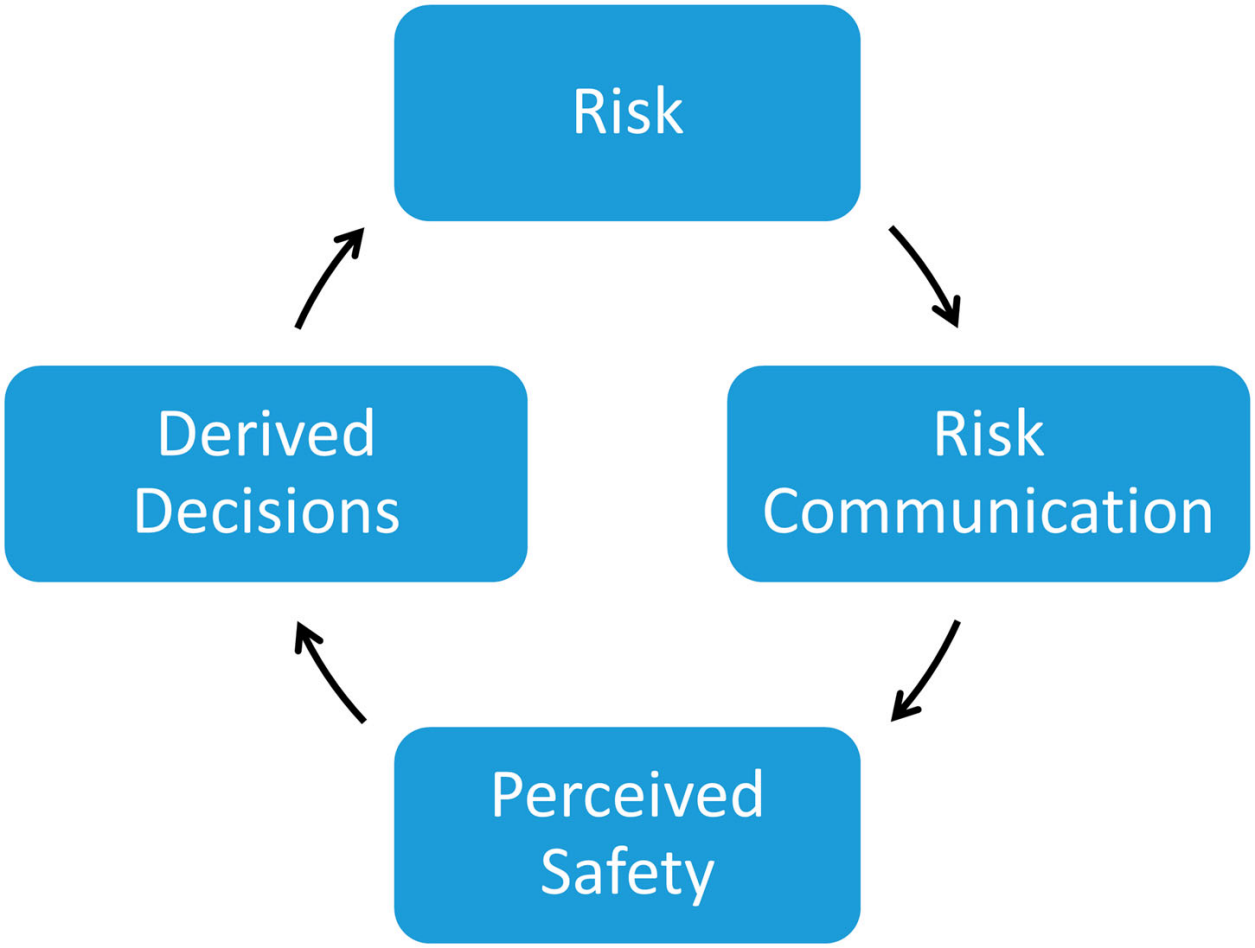
The Safety Loop. The safety loop describes the association and the mutual influence of an objective risk and the subjective perception of the objective risk (perceived safety). Objective risks can be assessed as the incidence of event times the size of damage (probability by noxiousness). The subjective perception of the objective risks can be described either by psychometric methods (supplement 1) or may be expressed by odds ratios (perceived safety or perceived anxiety) as described in this paper. Explanation of the safety loop: Existing risks trigger risk communication. The risk communication affects the subjective perception of objective risks. The subjective perception of the risk (perceived safety or anxiety) depends not only on communication but several factors (Porzsolt, 2016) that will govern the derived decision. The loop shows that a high-risk situation may emerge when the derived (subjective) decision has a strong effect on the initial objective risk and can potentially induce a self-containing process of a virtual risk. The true reason of this virtual risk is the validity of data that drives the subjective perception of the perceived safety and safety loop.

As our knowledge about SARS-CoV-2 increases High Reliability Organizations (HRO) should embrace the unexpected to successfully cope with hazards in sophisticated and complex systems (Marais, Dulac, & Leveson, 2004). The four recommendations to HROs require (a) recognition of the diﬀerence between reliability and safety, (b) training of members of HROs to provide appropriate responses to crisis situations, (c) usage of sophisticated forms of organizational learning, and (d) to use redundancy extensively. In line with those recommendations, a safety loop has been proposed (Figure 1). The ‘safety loop’ describes the interrelationship between objective risks, risk communication, the resulting subjective perception of objective risks, the derived consequences from the subjective perception of the objective risk and finally the effect of the decisions onto the objective risk (Porzsolt, Thomaz, Constâncio, Silva-Júnior, & Nóbrega, 2013, 2011).

In this paper, we describe the quantification of ‘Perceived Safety’ and ‘Perceived Anxiety’. The approach uses traditional 2 × 2 tables and combines this information with the Protection Motivation Theory (Rogers, 1975), the concept of High Reliability Organizations (Marais, Dulac, & Leveson, 2004), and the model of the Safety loop (Porzsolt, 2016) to propose a strategy – the information pyramid – on how to use scientific data for political decisions.

## Methods

### The examples used for application of the theory

We used data from 10 different scenarios to confirm our algorithm on the quantitative assessment of ‘Perceived Safety’ and ‘Perceived Anxiety’. Example #1 shows data from mammography screening reported by the Breast Cancer Surveillance Consortium (https://tools.bcsc-scc.org/BC5yearRisk/calculator.htm). The analysis considered expected outcomes for 5000 women aged 40 years, who are screened for breast cancer. Based on data from Carney and colleagues (Carney et al., 2003) it assumes the prevalence of breast cancer is 2 per 1000 with a mammography sensitivity of 0.66 and a specificity of 0.91. Examples #2–#4 show data on prostate cancer screening reported by Hugosson et al. (Hugosson et al., 2018) when using three different endpoints. Example #2 confirms the diagnosis of prostate cancer, example #3 the disease-specific mortality, and example #4 the all-cause mortality. Example #5 depicts data of a Bavarian study on mortality after myocardial infarction (Huml kuendigt fuer Bayern Schwerpunktkampagne zu Herzinfarkt an [press release], 2020). The examples #6–#9 are based on data reported by the Robert Koch Institute, Berlin on the Covid-19 Pandemic (Coronavirus SARS-CoV-2 – COVID-19: Fallzahlen in Deutschland und weltweit [press release], 2020). As these reports did not include sensitivity and specificity, we used four possible combinations of sensitivity and specificity for our calculations. Example #10 uses data from the Covid-19 Pandemic Norway database FHI (Norwegian Institute of Public Health) and a report in Norwegian TV (NRK, 2020).

#### The application of the theory

Our theory assumes that both the objective risks and the subjective perception of objective risks can be calculated from traditional 2 × 2 tables. The traditional 2 × 2 table starts with two hypotheses, e.g. a positive mammography confirms breast cancer while a negative mammography excludes breast cancer. For confirmation of the true diagnosis a gold standard, e.g. the histopathologic examination of the suspected lesion is necessary. The specimen can be collected by a fine needle biopsy. The 2 × 2 table enables the calculation of the positive and negative Likelihood Ratios (+LR; −LR). The + LR is the ratio of true positives over false positives. Similarly, the –LR is the ratio of false negatives over true negatives. A LR value of 1 means that the probability of confirmed (excluded) disease is identical in persons with a positive and with a negative test result. In other words, a test result with a LR = 1 is inconclusive. LR > 1, named + LR, can indicate the confirmation of a condition while LR < 1, named –LR, can indicate the exclusion of a condition. Further details for calculation and interpretation of LRs are described in Supplement 1.

The calculation of the subjective perception of the objective risks follows exactly the same rules as the calculation of the objective risks in a 2 × 2 table but with exchanged X- and Y-axes. The 2 × 2 table with inverted axes starts with the communication of the test result and presumes that the communication of bad news (the bad news may be true or false) can induce ‘Perceived Anxiety’ while good news (independently of being true or false) can induce ‘Perceived Safety’. The induction of perceived anxiety can be quantified by calculation of the LRs from an inverted table of a test that investigates bad news such as a diagnosis of cancer. A calculated +LR > 1 (−LR < 1) of a test that investigates the effects of bad news confirms (excludes) perceived anxiety. Correspondingly, a calculated +LR > 1 (−LR < 1) of a test that investigates the effect of good news such as prolongation of survival confirms (excludes) perceived safety.

## Results

The example shown in Table 1 uses data from the U.S. Breast Cancer Surveillance Consortium Task Force. The summaries of all Likelihood Ratios describing the confirmation or exclusion of the investigated endpoint and of the perceived anxiety and perceived safety are shown in Table 2.

**Table 1.** Example for calculations in traditional and inverted 2 × 2 table.

Traditional (afferent)	Breast Cancer Confirmed	Breast Cancer Not confirmed	Total
Mammogram positive	7	449	**456**
Mammogram negative	3	4541	**4544**
Total	**10**	**4990**	**5000**
**Sens: 0.70; Spec: 0.91**	+LR: 7.78	−LR: 0.33	Prevalence: 0.002
**New (efferent)**	Mammography Pos.	Mammography Neg.	Total
Breast Ca. Confirmed	7	3	**10**
Breast Ca. Not conf.	449	4541	**4990**
Total	**456**	**4544**	**5000**
	Perceived Anxiety: 23.25	Perceived Safety: 0.99	

Legend: Data for example #1 breast cancer screening reported by the Breast Cancer Surveillance Consortium tool (see https://tools.bcsc-scc.org/BC5yearRisk/calculator.htm) to estimate confirmation (or exclusion) of the suspected diagnosis by calculation of the positive (or negative) Likelihood Ratio derived from the traditional 2 × 2 table. The new version (with exchanged X- and Y-axes) of the same table are used for quantification of the Perceived Anxiety (or Perceived Safety) by estimating the positive (or negative) Likelihood Ratio.

**Table 2.** Likelihood Ratios of ten tests.

Line	Likelihood Ratios	Test #1 Br. Ca	Test #2 Pr. Ca.	Test #3 Pr. DSM	Test #4 Pr. ACM	Test #5 Mort MI.	Test #6 P-Ger 95/70	Test #7 P-Ger 90/70	Test #8 P-Ger 70/70	Test #9 P-Ger 70/99	Test #10 P-Nor 99/96
1	Confirm.	7.78	1.19	0.84	0.75	31.1	3.19	3.00	2.33	70.0	27.0
2	PERA	23.3	3.09	0.55	0.49	23.5	39.4	18.6	5.03	57.0	5891
3	Exclusion	0.33	0.35	1.54	2.21	0.95	0.07	0.14	0.43	0.30	0.97
4	PESA	0.99	0.91	1.01	1.45	0.78	0.88	0.89	0.92	0.25	.004

Legend: Tests #3 and #4 (blue background) describe a *wanted* effect whereas all other tests describe *not wanted* conditions The positive Likelihood Ratios describe the confirmation (+LR > 3) if *not wanted conditions* are investigated in line 1 and the Perceived Anxiety in line 2 (tests #1, #2, #5, #6, #7, #8, #9, and #10). In tests #3 and #4 wanted conditions are described. The −LRs < 1 in lines 1 and 2 express the direction of the test towards exclusion of the condition and the + LR > 1 in lines 3 and 4 express the direction of the test towards confirmation of the condition. The results of tests #3 and #4 can neither confirm nor exclude an investigated condition nor perception as none of the calculated LR did exceed the limits of the indifference zone.

Table 1 shows a fair reliability of the test for confirmation of the diagnosis of breast cancer (+LR = 7.78) but not for the exclusion of the diagnosis (−LR = 0.33). The inverse table in the lower part of Table 1 shows that the + LR is highly reliable to confirm considerable ‘Perceived Anxiety’: +LR = 23.2 while it cannot exclude ‘Perceived Safety’: −LR = 0.99. The corresponding data from additional nine scenarios are shown in Supplement 2.

Table 2 summarizes the results of all ten scenarios. Line 1 shows the + LR signaling confirmation, line 3 the –LR signaling exclusion (traditional 2 × 2 table analysis). Line 2 reports the inverted calculations, here the + LR signals ‘Perceived Anxiety’ (PERA; line 2), and line 4 the –LR signals ‘Perceived Safety’ (PESA; line 4).

Two scenarios, #3 and #4, describe a wanted effect, here the reduction of Disease-Specific Mortality (DSM) or of All-Cause Mortality (ACM). All other tests describe undesirable conditions such as evidence for Breast Cancer (Br.Ca), Prostate Cancer (Pr.Ca), Mortality following myocardial infarction (mort MI), or Positive Polymerase Chain Reaction German Test or Norwegian Test including sensitivity and specificity.

In line 1 eight of the ten tests show a +LR > 1 but two tests (tests #3 and #4) show a −LR < 1. The positive LRs confirm the investigated conditions while the negative LRs exclude the investigated condition. To understand the results of 2 × 2 tables it is necessary to consider the valence of confirmation (good news or bad news) for correct interpretation of the results. The eight tests that generated + LR assumed bad news, e.g. death or infection whereas the two tests (test #3 and #4) that generated −LR, expressed good news i.e. reduction of mortality.

Line 1 of Table 2 shows that three tests (test #5, #9, #10) confirm very likely (+LR > 10) the investigated conditions, two additional tests (test #1 and #6) confirm likely (+LR > 3) the investigated condition, and five tests (tests #2, #3, #4, #7, and #8) can neither confirm nor reject the investigated condition because their LRs do not exceed the likelihood indifference zone (LRs between 0.3 and 3). The three tests (tests #2, #7, #8) with +LR > 1 failed to confirm the investigated condition and two tests (tests #3 and #4) with −LR > 0.3 failed to exclude the investigated condition.

Line 2 of Table 2 describes that eight of the ten tests result in increased ‘Perceived Anxiety’. One of these tests (test #10) shows a very high probability of increased ‘Perceived Anxiety’: +LR > 3000. Five tests (test #1, #5, #6, #7, #9) confirm very likely the induction of ‘Perceived Anxiety’: +LR > 10. Two additional tests (tests #2 and #8) confirm moderately the induction of ‘Perceived Anxiety’: +LR > 3, but two other tests (test #3 and #4) fail to exclude the induction of ‘Perceived Anxiety’: −LRs of 0.55 and 0.49, respectively.

Line 3 of Table 2 shows that a −LR < 0.3 that would be strong enough to exclude the investigated condition, was observed in only two of the ten tests (test #6 and #7). These two tests exclude the presumed correlation of PCR with viral disease.

Line 4 shows that two of the ten tests exclude the induction of ‘Perceived Safety’ (PESA) (i.e. test #9 and #10). The remaining eight tests can neither confirm nor exclude the perception of safety. In other words, a negative test result does not reassure the participant.

The LRs in Table 2 also demonstrate that the traditional tables (description of cases detected) and the inverted tables (description of the induced psychological perception) generate different and independent results.

**Figure 2.**
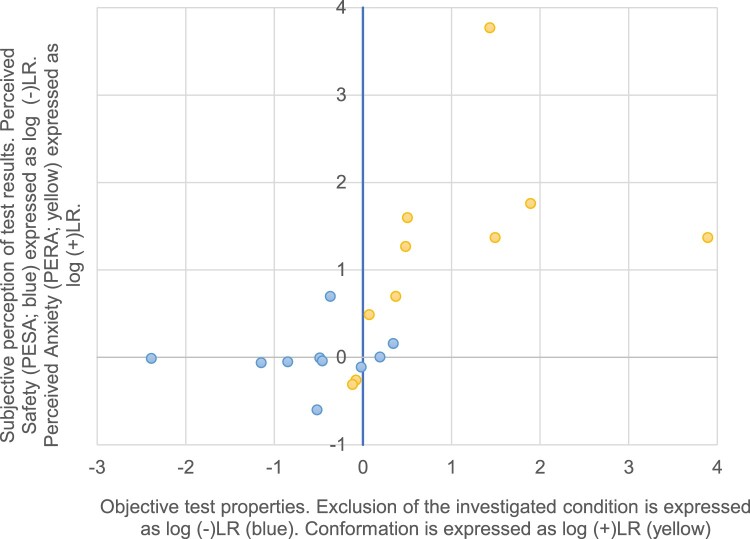
Correlation of the objective functions (X-axis expressed as exclusion or confirmation) of tests and the subjective perception of the objective functions (Y-axis expressed as Perceived Safety or Perceived Anxiety) caused by these tests.

Figure 2 provides a logarithmic presentation of the data. It shows the correlation between the objective functions (X-axis expressed as exclusion or confirmation) of tests and the subjective perception of the objective functions (Y-axis expressed as ‘Perceived Safety’ or ‘Perceived Anxiety’). Most tests cannot exclude (blue points on X-axis) but can confirm (yellow points on X-axis) a diagnosis. Accordingly, most of our investigated tests cause ‘Perceived Anxiety’ (PERA) but not ‘Perceived Safety’ (PESA).

## Discussion

Our results demonstrate that each 2 × 2 test table has two independent sets of functions. The first set of (traditional) functions describes the correlation of a test result with a confirmed diagnosis. The second set of (new) functions describes the correlation of the confirmed diagnosis with the generation of psychological effects. Our results also show that any inappropriate interpretation of a test result will induce harmful effects that may cause – via an incorrect diagnosis – an inappropriate treatment of a single patient (Newman-Toker et al., 2021) or may cause – via inappropriate political decisions – a national or even international policy failure (e.g. Todd (2020), Moon (2020)).

Tests describe only probabilities, never a certainty. Due to the strong psychological effects that can be caused by inappropriate interpretation of test results, it is important to distinguish between professional consideration and public communication of test results and their interpretations. Some examples may be useful to elucidate the ‘explosive force’ of test results.

At the individual level, there are recommendations for physicians and genetic counselors on communicating uncertainties to their patients (Medendorp et al., 2021; Stivers & Timmermans, 2016; Trevena et al., 2013; Wood, Prior, & Gray, 2003; Zhong, Donovan, & Vangelisti, 2021) and thereby helping them to cope with the uncertainty. This will also facilitate a representation of the illness experience that leads to appropriate actions and health behavior and appraisal or emotional response (Hale, Treharne, & Kitas, 2007). This common sense model of self-regulation of health and illness (Leventhal et al., 2012) suggests that health-and illness-related decisions are impacted by lay understandings of the symptoms, labels, causes, time-line, consequences, and controllability of the disease and one's health. Emotions and illness representations influence each other and interact to affect coping responses and health behavior decisions. Accordingly, perceived health threats, in our case the risk of COVID-19 infection might result in both emotional and cognitive manifestations that influence both coping resources and health behavior (Cameron & Jago, 2008; Helsingen et al., 2020; Lammers, Crusius, & Gast, 2020; Mækelæ et al., 2020, 2021; Sibley et al., 2020; Torales, O’Higgins, Castaldelli-Maia, & Ventriglio, 2020; Varga et al., 2021). Of the five components of the illness representation, controllability ascribes an active role to the individual. The communication should be adapted to individual preferences, beliefs and coping styles, as well as provide a sense of control, hope and emotional support (Medendorp et al., 2021). Communicating health threats can lead to negative emotions, which in turn can be reduced with reappraisal techniques without affecting preventive health behaviors (Wang et al., 2021).

**Figure 3.**
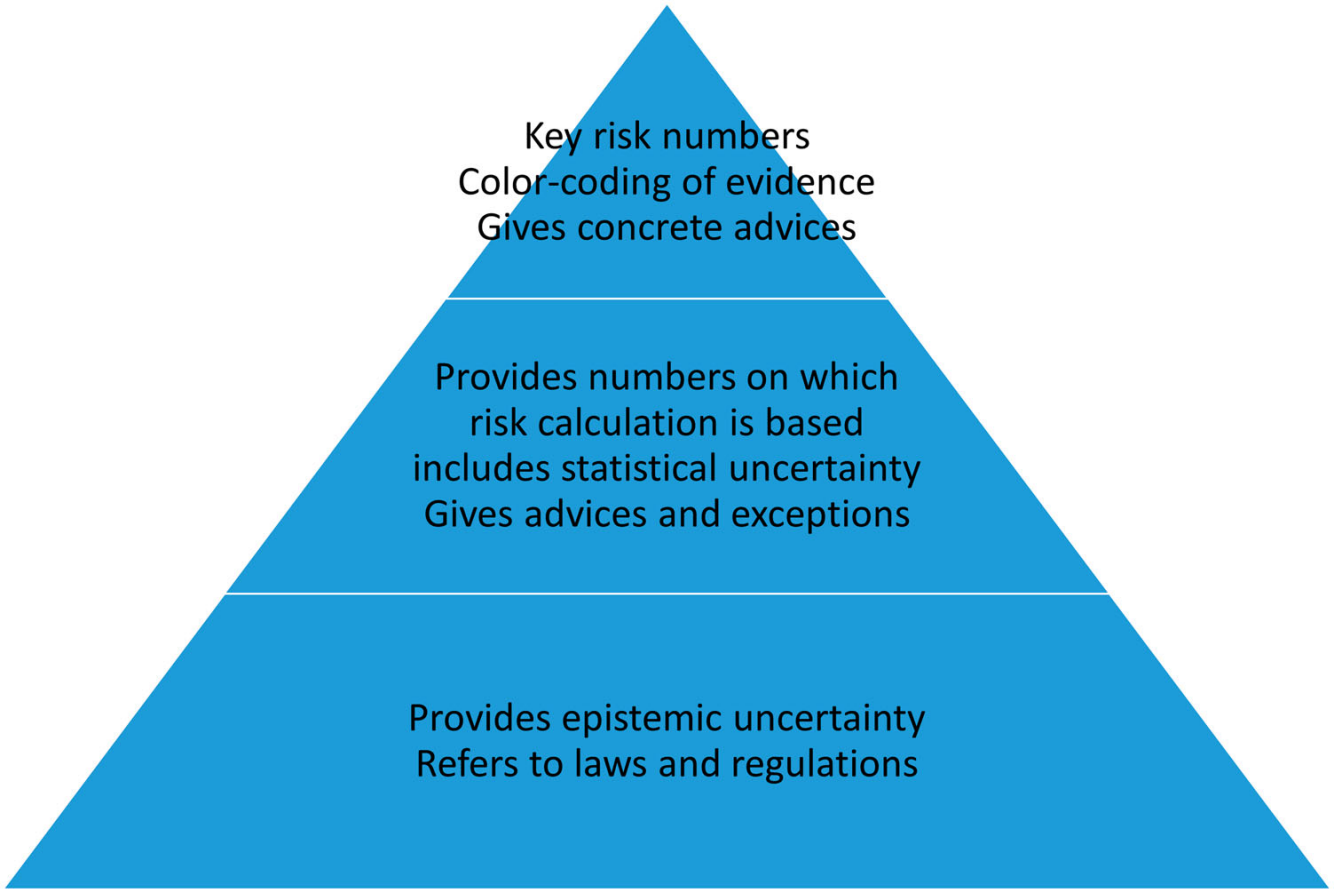
Information pyramid. For uncertain and complex information, the balance between transparency and clarity is to provide the amount of information hierarchically. At the top should be the key message(s), e.g. the likelihood ratios; followed at the second level by providing the confusion matrix and explaining the derivation, including statistical uncertainty. The third level also provides model uncertainty and assumptions. Decision- and policy-makers should start at the bottom to derive valid key messages and concrete advices which are then presented at the top.

Importantly, risk communication in emerging diseases and pandemics has to be different from risk communication of chronic health issues, genomic results, or natural hazards (Piltch-Loeb & Abramson, 2020). To address and control an emerging threat, both individual-level interventions and policy-oriented interventions have to be implemented (Piltch-Loeb & Abramson, 2020). At the individual level, these are for example quarantine, mask wearing and hand washing. At the community level, these are for example closing of schools, border closures and limiting mass gatherings. Further, the knowledge growth during a pandemic requires a dynamic, transparent communication (e.g. Baker, Wilson, and Anglemyer (2020), McGuire, Cunningham, Reynolds, and Matthews-Smith (2020)). The challenge is to communicate adequately stochastic and epistemic uncertainty, without leading to e.g. confusion, distress or defiance (e.g. Mækelæ et al. (2021), Serafini et al. (2020), Fitzpatrick, Drawve, and Harris (2020)). To promote intervention acceptance it is therefore paramount to understand the threat and the perceived risk of the threat (Lindell & Perry, 2012; Mækelæ et al., 2020). In case of emerging threats (pandemics), statistical tests not only convey stochastic uncertainty but also epistemic uncertainty (Han, Klein, & Arora, 2011). To facilitate transparent communication of uncertain information and providing behavior recommendations – both at the individual and the community level – we suggest to apply an information pyramid (Figure 3). At the top key numbers and advice should be presented. In the case of communicating the results of a confusion matrix, these are the odds ratios. They might be color-coded if they are above 3 or below 1/3. Behavioral recommendations and rules should be provided in an autonomy-supportive way (Legate & Weinstein, 2021). At the second level, data from which the key numbers are derived should be presented, e.g. the confusion matrix. At the third level, the assumptions and model uncertainty underlying the numbers are presented and explained. The information pyramid should use graphical and numerical representation, ensuring both transparency and ease of understanding, thereby allowing informed decision-making tailored to the needs and capacities of an individual.

Gerd Gigerenzer and colleagues have published material for experts and ‘ordinary people’ to guide interpretation of numbers, tests, probabilities, and conditional probabilities (Gigerenzer & Gaissmaier, 2011; Gigerenzer & Gray JA, 2013) – helpful for understanding stochastic uncertainties. There is no universal recommendation for communicating epistemic uncertainty. People may respond to disclosing it with more or less trust to authorities, and hence with engaging in more or less protective behavior (Ratcliff, Wong, Jensen, & Kaphingst, 2021).

We strongly advise to consider the uncertainty in the interpretation of test results (Engeset, Pfuhl, Landrø, Mannberg, & Hetland, 2018; Medendorp et al., 2021; Pearce, 2020; Stivers & Timmermans, 2016; van der Bles et al., 2019). Expert knowledge has improved, and new research will continue to modify test interpretation. It is often better to admit uncertainties than to provide inconsistency as these reduce governmental credibility (Moon, 2020; Rafkin, Shreekumar, & Vautrey, 2021; Ratcliff et al., 2021). We also recommend to provide all available information to avoid a biased risk perception (Abel et al., 2021) and increase the knowledge about a threat and illness representation, respectively (Hale et al., 2007).

As shown, understanding the statistical principles underlying diagnostic tests is the first of two necessary steps. The second step is the derivation of correct decisions based on test results. The correct derivation of practical consequences requires different knowledge than the correct interpretation of statistical test results (Haaland, Roth, & Wohlfart, 2020). The interaction between preferences and faulty inference can lead to negative economic consequences (Eil & Rao, 2011; Moon, 2020).

The interface between medicine and public policy remains challenging. Medical professionals are usually not trained in public policy, and policy makers are rarely qualified medical doctors. We may need a new professional group that can bridge medicine and politics (Pearce, 2020).

Controversial discussions of results are essential in science but may be disturbing for political decisions. Investigative journalists might get advanced training in the communication of conflicting scientific perspectives and the interplay between preferences, constrains and beliefs. They should identify the published peer-reviewed knowledge, to exclude immature considerations and opinions, to moderate the controversial scientific discussion on solid data, and finally extract the currently reliable state of the art from a dynamic scientific process. This process could be open to the public and may immediately be available for the policy discussions. The goal of any consequences and the assessed endpoints and time intervals should be defined to restore the lost confidence in the policy decision process.

## Supplementary Material

Supplemental Material
