# Supplementary material for: Covid-19 pandemic lessons: uncritical communication of test results can induce more harm than benefit and raises questions on standardized quality criteria for communication and liability
Source: Health Psychol Behav Med. 2021 Sep 21;9(1):818–29. doi: 10.1080/21642850.2021.1979407 (PMC8462930; doi:10.1080/21642850.2021.1979407)
Supplement: Supplemental Material [file RHPB_A_1979407_SM6225.docx]

**Supplement I: Examples for calculation of the +LR & -LR and of PERA & PESA**

The upper part of the example explains the calculation of +LR & -LR. The lower part of the example shows the exchanged X- and Y-axes of the same table as above. By using the version of the table with exchanged axes the calculation of PERA & PESA can be completed by the same strategy that was used for calculation of the +LR & -LR.
The result of the (+)LR describes the x-fold excess of the frequency of a positive test in the group with the conditions (e.g. in women with biopsy confirmed breast cancer) than in the group without this condition (i.e. no biopsy confirmed breast cancer).
Please remember each test has two different types of qualities. A test can either confirm an assumption of an *unwanted condition* or reject it or both ore none. The +LR confirms an unwanted condition. +LR = 5 means the chance to find a test-positive person among those with the unwanted condition will be 5-times higher than the chance to find a test-positive person among those without this condition.
LR = 1 means neither confirmation nor exclusion. The closer the LR is to the value of 1 the higher is the risk of this test neither to confirm nor to exclude. Therefore, many researchers consider results in the zone around LR = 1, i.e. between a -LR of 0.3 and a +LR of 3.0 a zone of indifference. -LR between 0.3 and 0.1 can be considered fairly good to exclude a condition and those smaller than 0.1 can almost certainly exclude a condition. Corresponding results apply to +LR. +LR between 3.0 and 10.0 may fairly well confirm a condition and values above 10.0 almost certainly confirm a condition.

We are using the example of breast cancer to demonstrate that the quality of the ‘Gold Standard’ may vary depending on the type of the selected ‘Gold Standard’. One of the excellent Gold Standards for breast cancer is the confirmation of advanced stage of disease because even biopsy confirmed breast cancer may disappear spontaneously (Kaplan et al. ; Porzsolt et al.) or will not be detected by mammography in 3 of 10 cases (see supplement II). Such residual uncertainties should be considered in any type of test. The following 2 x 2 table is a standard version for confirmation of both LRs:

|  | Condition met | Condition unmet | Σ |
| --- | --- | --- | --- |
| Test + | A | B | A + B |
| Test - | C | D | C + D |
| Σ | A + C | B + D | A+ B + C + C + D |

The formula for confirmation of an unwanted condition. +LR = [A / (A + C)] : [B / (B + D)].
The formula for exclusion of an unwanted condition. -LR = [C / ( A + C)] : [D / (B + D)].
The corresponding table with exchanged X- and Y-axes for calculation of PERA & PESA is:

|  | Test + | Test - | Σ |
| --- | --- | --- | --- |
| Condition met | A | C | A + C |
| Condition unmet | B | D | B + D |
| Σ | A + B | C + D | A+ B + C + C + D |

The formula for confirmation of the effect of the unwanted condition i.e. Perceived Anxiety (PERA) is
+LR = [A / (A + B)] : [C / (C + D)].
The formula for exclusion of the effect of the unwanted condition i.e. Perceived Safety (PESA)is
-LR = [B / (A + B)] : [D / (C + D)]

**Supplement II:**

Examples #2 - #10. The following tables show the raw data and calculated values summarized in Tab. 2 of the related publication.

Example #2 Prostate Cancer Screening (Endpoint Cancer) (Ref: Hugosson)

| **Traditional (afferent)** | Prostate Ca. Conf. | Prostate Ca. Not conf. | Total |
| --- | --- | --- | --- |
| Accepted screening | 1272 | 6575 | **7647** |
| Rejected screening | 124 | 2179 | **2302** |
| Total | **1396** | **8554** | **9950** |
| **Sens: 0.91; Spec: 0.25** | Pos. LR: 1.185 | Neg. LR: 0.349 | Prevalence: 0.77 |
| **New (efferent)** | Accepted Screening | Refused Screening | Total |
| Prostate Ca. Conf. | 1272 | 124 | **1396** |
| Prost. Ca. Not conf. | 6575 | 2179 | **8554** |
| Total | **7647** | **2302** | **9950** |
|  | Perceived Anxiety PERA: 3.088 | Perceived Safety  PESA: 0.908 |  |

Example #3 Prostate Cancer Screening (Endpoint Disease Specific Mortality) (Ref: Hugosson)

| **Traditional (afferent)** | Died of Prostate Ca. | Not died of Prost. Ca. | Total |
| --- | --- | --- | --- |
| Accepted screening | 51 | 7596 | **7647** |
| Rejected screening | 28 | 2275 | **2303** |
| Total | **79** | **9871** | **9950** |
| **Sens: 0.65; Spec: 0.23** | Pos. LR: 0.84 | Neg. LR: 1.54 | Prevalence: 0.77 |
| **New (efferent)** | Accepted Screening | Refused Screening | Total |
| Died of Prostate Ca. | 51 | 28 | **79** |
| Not died of Prost. Ca. | 7596 | 2275 | **9871** |
| Total | **7647** | **2303** | **9950** |
|  | Perceived Anxiety PERA: 0.55 | Perceived Safety PESA: 1.01 |  |

Example #4 Prostate Cancer Screening (Endpoint All-cause Mortality) (Ref: Hugosson)

| **Traditional (afferent)** | Dead (any cause) | Alive | Total |
| --- | --- | --- | --- |
| Accepted screening | 1763 | 5884 | **7647** |
| Rejected screening | 1081 | 1222 | **2303** |
| Total | **2844** | **7106** | **9950** |
| Sens: 0.62; Spec: 0.17 | Pos. LR: 0.75 | Neg. LR: 2.21 | Prevalence: 0.77 |
| **New (efferent)** | Accepted Screening | Refused Screening | Total |
| Dead (any cause) | 1763 | 1081 | **2844** |
| Alive | 5884 | 1222 | **7106** |
| Total | **7647** | **2303** | **9950** |
|  | Perceived Anxiety PERA: 0.49 | Perceived Safety PESA: 1.45 |  |

Example #5 Survival following Myocardial Infarction (MI) x10E5 (Ref: Bavarian Ministry Social Affairs)

| **Traditional (afferent)** | Heart related death | Death other reason | Total |
| --- | --- | --- | --- |
| With MI | 0.069 | 0.231 | **0.3** |
| Without MI | 1.27 | 128.43 | **129.7** |
| Total | **1.34** | **128.66** | **130.0** |
| **Sens: 0.05; Spec: 1.00** | Pos. LR: 28.68 | Neg. LR: 0.95 | Prevalence: 0.002 |
| **New (efferent)** | With MI | Without MI | Total |
| Heart related death | 0.069 | 1.27 | **1.34** |
| Death other reason | 0.231 | 128.43 | **128.66** |
| Total | **0.300** | **129.70** | **130.00** |
|  | Perceived Anxiety PERA: 23.5 | Perceived Safety PESA: 0.78 |  |

Example #6 Pandemic PCR-Test. (Ref: Robert Koch Institute Berlin / Germany)

| **Traditional (afferent)** | Disease Spec. Mortal. | Alive | Total |
| --- | --- | --- | --- |
| Pat. with positive test | 6646 | 46993 | **53639** |
| Without positive test | 350 | 110818 | **111168** |
| Total | **6996** | **157811** | **164807** |
| **Sens: 0.95; Spec: 0.70** | Pos. LR: 3.19 | Neg. LR: 0.07 | Prevalence: 0.33 |
| **New (efferent)** | Pat with positive test | Pat without pos. test | Total |
| Disease Spec. Mortal. | 6646 | 350 | **6996** |
| Alive | 46993 | 110818 | **157811** |
| Total | **53639** | **111168** | **164807** |
|  | Perceived Anxiety PERA: 39.4 | Perceived Safety PESA: 0.88 |  |

Example #7 Pandemic PCR-Test. (Ref: Robert Koch Institute Berlin / Germany)

| **Traditional (afferent)** | Disease Spec. Mortal. | Alive | Total |
| --- | --- | --- | --- |
| Pat. with positive test | 6296 | 47343 | **53639** |
| Without positive test | 700 | 110468 | **111168** |
| Total | **6996** | **157811** | **164807** |
| Sens: **0.90; Spec: 0.70** | Pos. LR: 3.00 | Neg. LR: 0.14 | Prevalence: 0.33 |
| **New (efferent)** | Pat with positive test | Pat without pos. test | Total |
| Disease Spec. Mortal. | 6296 | 700 | **6996** |
| Alive | 47343 | 40468 | **157811** |
| Total | **53639** | **111168** | **164807** |
|  | Perceived Anxiety PEAN: 18.6 | Perceived Safety PESA: 0.89 |  |

Example #8 Pandemic PCR-Test. (Ref: Robert Koch Institute Berlin / Germany)

| **Traditional (afferent)** | Disease Spec. Mortal. | Alive | Total |
| --- | --- | --- | --- |
| Pat. with positive test | 4897 | 47343 | **52240** |
| Without positive test | 2099 | 110468 | **112567** |
| Total | **6996** | **157811** | **164807** |
| **Sens: 0.70; Spec: 0.70** | Pos. LR: 2.33 | Neg. LR: 0.43 | Prevalence: 0.32 |
| **New (efferent)** | Pat with positive test | Pat without pos. test | Total |
| Disease Spec. Mortal. | 4897 | 2099 | **6996** |
| Alive | 47343 | 110468 | **157811** |
| Total | **52240** | **112567** | **164807** |
|  | Perceived Anxiety PEAN: 5.03 | Perceived Safety PESA: 0.92 |  |

Example #9 Pandemic PCR-Test. (Ref: Robert Koch Institute Berlin / Germany)

| **Traditional (afferent)** | Disease Spec. Mortal. | Alive | Total |
| --- | --- | --- | --- |
| Pat. with positive test | 4897 | 1578 | **6475** |
| Without positive test | 2099 | 156233 | **158332** |
| Total | **6996** | **157811** | **164807** |
| **Sens: 0.70; Spec: 0.99** | Pos. LR: 70.0 | Neg. LR: 0.30 | Prevalence: 0.04 |
| **New (efferent)** | Pat with positive test | Pat without pos. test | Total |
| Disease Spec. Mortal. | 4897 | 2099 | **6996** |
| Alive | 1578 | 156233 | **157811** |
| Total | **6475** | **158332** | **164807** |
|  | Perceived Anxiety PEAN: 57.0 | Perceived Safety PESA: 0.25 |  |

Example # 10 Pandemic PCR-Test. (Ref: **FHI (May 16,2020)** & NRK via Arctic University of Tromsø/Norway)

| **Traditional (afferent)** | Disease Spec. Mortal. | Recovered | Total |
| --- | --- | --- | --- |
| Pat. with positive test | **232** | 7965 | **8197** |
| Without positive test | 1 | 208152 | 208153 |
| Total | 233 | 216117 | **216350** |
| **Sen: 0.996 Spec: 0.963** | Pos. LR: 27.0 | Neg. LR: 0.004 | Prevalence: 0.038 |
| **New (efferent)** | Pat with positive test | Pat without pos. test | Total |
| Disease Spec. Mortal. | 232 | 1 | 233 |
| Recovered | 7965 | 208152 | 216117 |
| Total | **8197** | 208153 | **216350** |
|  | Perceived Anxiety PERA: 5891.3 | Perceived Safety PESA: 0.97 |  |
